# Dispositional mindfulness in climbers with different levels of experience

**DOI:** 10.1038/s41598-025-26202-x

**Published:** 2025-11-04

**Authors:** Petra Jansen, Hanna Wolters

**Affiliations:** Faculty of Human Science, University of Regensburg, Am Biopark 12, 93053 Regensburg, Germany

**Keywords:** Climbing, Risk-sports, Mindfulness, Emotion-regulation, Human behaviour, Risk factors

## Abstract

This cross-sectional study investigated whether climbing experience is associated with higher levels of dispositional mindfulness and its related key mechanisms. A total of *N* = 203 climbers—comprising 33 leisure and novice climbers, 85 moderately experienced climbers, and 85 experienced climbers—primarily from Germany (113 women, 86 men, and three non-binary individuals, aged between 20 and 61 years) completed five self-report questionnaires to test the hypotheses concerning *mindfulness* (measured with the Five Facet Mindfulness Questionnaire)*, attention* regulation (measured with the Attention Control Scale), and *emotion regulation* (measured with the Brief Version of the Difficulties in Emotion Regulation Scale*)* across varying levels of climbing experience. In addition, the study examined the relationship between *climbing experience* and *body awareness* (measured with the Embodied Mindfulness Questionnaire), and *non-attachment* (measured with the Short Form of the Non-Attachment Scale). Experienced climbers scored higher than moderately experienced climbers on the *non-judging* facet of dispositional mindfulness. In contrast, moderately experienced climbers reported significantly higher overall values of *emotion regulation* in the sub-scales of *clarity*, *strategies,* and *non-acceptance* compared to experienced climbers. Furthermore, the climbing and meditation experience was associated with attention to and awareness of bodily sensations. To conclude, the experience of climbing is only related to the non-objective facet of dispositional mindfulness. The observed advantages in emotion regulation among experienced climbers suggest that sustained engagement in climbing is associated with an agentic emotional experience.

## Introduction

The body of research on the relationship between sports activity and mindfulness is considerably smaller than the broader field of mindfulness research^1^. It is essential to distinguish between dispositional mindfulness, which refers to mindfulness as a stable individual characteristic or trait^2^, and mindfulness cultivated through interventions such as the 8-week Mindfulness-Based Stress Reduction (MBSR) program^3^. Mindfulness-based interventions have increased dispositional mindfulness, and long-term meditators tend to exhibit higher levels of dispositional mindfulness^4–6^. The fundamental mechanisms by which mindfulness interventions^7^ increase dispositional mindfulness primarily involve self-regulatory processes that interact with each other, namely: (a) *attention regulation*, (b) *emotion regulation*, and (c) *self-awareness* (including awareness of moment-to-moment experiences and self-referential processing). Those key mechanisms are also central processes in mindfulness research within sports contexts^1^. Attention regulation generally encompasses three aspects: alerting, orienting, and conflict monitoring (or executive attention)^8^. Emotion regulation, the second key mechanism, refers broadly to strategies to modify emotional responses^9^. Effective emotion regulation is crucial for human experiences, behavior, well-being, and mental health^10^. Tang et al.^8^ identify self-awareness as the third key mechanism of mindfulness, characterized by an enhanced awareness of moment-to-moment experiences and reduced self-referential processing. There is a gap in research regarding whether climbers exhibit a high manifestation of the trait of mindfulness.

As mentioned above, attention regulation, emotion regulation, and self-awareness are highly relevant in the context of sport: Attentional regulation, for example, is known to be influenced by sports and exercise^11,12^ (though see also for a non-significant difference)^13^. For athletes, adaptive emotion regulation involves various active cognitive and behavioral processes that enable effective responses to environmental demands^9^. Regarding the third key mechanism, self-awareness, a review article by Wallmann-Jones et al.^14^ suggests that exercise enhances interoception, particularly through mindful movement practice such as yoga^14–16^. Self-referential processing, which connects experiences to one’s person, is a critical feature across various concepts of the self, encompassing the processing and evaluation of both physical and psychological stimuli^17,18^.

One specific type of sport, rock climbing, is a broad term encompassing several recreational and competitive disciplines, such as bouldering (without a rope relying on protective mats), lead climbing (the climber is attached to a rope that is clipped into a series of bolt anchors), and top-roping (the safety rope passes through a top anchor and returns to a belayer^19–21^. It has been related to various cognitive and affective psychological concepts^22^. Climbers also have the option to practice their sport outdoors on different rock types^23^. Some forms of rock climbing, such as free soloing, are classified as high-risk sports; however, the injury rate for indoor climbers is considered, at worst, moderate^24^.

Regarding attention regulation in climbers, Heilmann^25^ investigated executive function in a small sample of sport climbing novices and experts but did not find differences in performance on an attentional task. Garrido-Palomino et al.^26^ examined the attentional skills of elite climbers, using a different attentional task, and found that higher on-sight climbing performance was associated with better attentional control. They suggested that enhanced attentional control is a crucial component of climbing performance. Given that climbing often involves high-risk situations, climbers require several psychological strengths, including a high level of attentional focus^27^ and the ability to accurately evaluate their mental and physical skills and expertise^28^.

According to the Agentic Emotion Regulation Theory^29^, high-risk sports such as climbing provide individuals with a sense of emotional control, as the source of anxiety is concrete and manageable within the activity context^30,31^. These identifiable stressors enable athletes to employ effective emotion-regulation strategies, transforming climbing and mountaineering into tools for emotional regulation^32–34^. With increasing experience and skill level, climbers reported fewer emotion-driven thoughts, indicating a shift in attentional focus away from anxiety and toward performance-related cues^35^. This reduction in emotional interference is associated with enhanced climbing performance.

Furthermore, evidence suggests that frequent exposure to risk positively influences the everyday functioning of high-risk athletes, contributing to improved emotion regulation and higher self-esteem^36^. Experienced climbers, in particular, are frequently placed in situations that require them to manage intense emotional responses, providing consistent opportunities to develop and refine their emotion-regulation skills. Additionally, climbers often score high on sensation-seeking—a personality trait characterized by pursuing novel, complex, and intense experiences, even when such pursuits involve financial, social, or physical risks. This trait motivates engagement in high-risk sports and supports the development of adaptive psychological skills through repeated exposure to risk^37^.

Dispositional mindfulness in athletes has been investigated only rarely. Findings from one study suggest that conscientious and emotionally stable athletes are more aware of the present moment and can adopt a nonjudgmental attitude, two components of dispositional mindfulness^38^. In another study, the relationship between mindfulness, both as dispositional mindfulness and as the result of a short intervention, and rock climbing was examined^39^: A brief mindfulness intervention followed by indoor bouldering led to a significant increase in dispositional mindfulness compared to a control group that received a physical activity intervention. However, no significant effects were found for the measures of well-being and anxiety.

The present study builds on the work of Cebolla et al.^40^, who assessed and compared dispositional mindfulness and its key mechanisms (in their research, in contrast to the study of Tang et al.^8^, *attention control, emotion regulation, non-attachment*, *non-reactivity*, and *body awareness*) in novice and expert meditators using a cross-sectional design. Their findings suggest regular meditation enhances attentional control, body awareness, emotion regulation, and non-attachment. Non-attachment refers to maintaining well-being without overly relying on external objects and detaching from ego-driven desires. In sports, non-attachment and dispositional mindfulness are essential for athletes’ well-being^41^. Research by Lewis et al.^41^, involving athletes from various disciplines, found that non-attachment had a more substantial positive effect on well-being than mindfulness alone. Furthermore, non-attachment may be a key mediating factor in the relationship between mindfulness and athlete burnout, suggesting its protective role in maintaining psychological health in high-performance environments^42^.

Accordingly, this study investigated the levels of mindfulness and its associated key mechanisms among climbers with varying levels of experience, employing a cross-sectional design. The primary aim was to determine whether long-term participation in climbing is associated with differences in the key mechanism of dispositional mindfulness, as studied by Cebolla et al.^40^. To address this aim, the following hypotheses were formulated:

1. Based on previous findings indicating that psychological attributes associated with mindfulness, such as attentional control and emotion regulation, tend to be more developed in experienced athletes, it is hypothesized that more experienced climbers will exhibit higher levels of dispositional mindfulness than novice or less experienced climbers.
2. Given that attention regulation is positively influenced by participation in sport and exercise, it is hypothesized that more experienced climbers will demonstrate higher levels of attentional control than less experienced climbers.
3. In line with the Agentic Emotion Regulation Theory and considering that experienced climbers have more opportunities to develop emotional regulation skills through repeated exposure to challenging situations, it is hypothesized that more experienced climbers will exhibit better emotion regulation than less experienced climbers.
4. The relationship between body awareness, non-attachment, and various individual factors will be investigated in an exploratory manner. Specifically, body awareness will be examined in relation to climbing experience, meditation experience, age, gender, and the interaction between meditation and climbing experience. The relationship between meditation experience and body awareness has been previously established^40^. Additionally, the relationships between body awareness, climbing experience, age, and gender are also considered as possible important correlational factors. The other key mechanism of dispositional mindfulness, according to Cebolla et al.^40^, namely non-reactivity, was already examined in Hypothesis 1 as a facet of mindfulness.

## Method

### Participants

Before data collection, power analyses were conducted for the four hypotheses using the software G*Power (Version 3.1.9.7^43^, see supplementary material). The highest required sample size emerged for the second hypothesis: assuming a medium effect size (*f* = 0.25), an alpha-level of *α* = 0.05, and a desired power of 1-ß = 0.80, power analysis for an ANOVA with four groups indicated a required sample size of *N* = 180 to detect significant differences in attentional control.

Of the 1018 participants who opened the survey link and began the questionnaire, 210 completed the entire survey (completion rate: approximately 21%). Several exclusion criteria were applied: a) Participants who did not complete any of the questionnaires were excluded (n = 3), b) Participants who rated their English proficiency as not good (“I do not understand the English language”) or “not so good” (“I had considerable difficulties in understanding”), were excluded (n = 3), c) Participants with unusual fast response times, indicated by a “degree_time variable” above 200, were excluded (n = 1). After exclusions, the final sample consisted of N = 203 participants (113 women, 86 men, and three diverse participants; one participant did not answer). Participants’ ages ranged from 20 to 61 years (M = 31.37, *SD* = 8.37). In terms of climbing experience, the largest group (n = 37, 18.2%) reported more than 10 years of climbing experience, followed by 26 participants (12.8%) with one to two years of experience and 25 participants (12.3%) with less than one year of experience. Demographic and climbing experience details are presented in Tables 1 and 2.

**Table 1:** Demographic data of the participating climbers.

Experience in years (*M, SD*)	Leisure & novice (N = 33) (*M* = 1.76, *SD* = .435)	Moderately experienced (N = 85) (*M* = 4.31, *SD* = 1.10)	Experienced (N = 85) (*M* = 10.08, *SD* = 2.03)
Age *M* (*SD*)	27.03 (5.89)	28.95 (4.83)	35.48 (10.08)
Gender			
Women	22 (66.7%)	49 (57.6%)	42 (49.4%)
Men	11 (33.3%)	35 (41.2%)	40 (47.2%)
Non-Binary		1 (1.2%)	2 (2.4%)
Not-Specified			1 (1.2%)
Mental Illness			
No	28 (84.8%)	68 (80%)	66 (77.6%)
Yes	5 (15.2%)	17 (20%)	19 (22.4%)
Native Language			
German	29 (87.9%)	69 (81.2%)	66 (77.6%)
English	1 (3.0%)	5 (5.9%)	3 (3.5%)
Other	4 (12.1%)	14 (16.5%)	17 (20.0%)
English Level			
Very good	25 (75.8%)	64 (75.3%)	58 (68.2%)
Good	8 (24.2%)	16 (18.8%)	221 (25.9%)
Okay		5 (5.9%)	5 (5.9%)
Meditation			
Never meditated	2 (6.1%)	11 (12.9)	11 (12.9)
Tried it once	7 (21.2%)	27 (31.8%)	28 (32.9)
Couple of times a year	4 (12.1%)	21 (24.7%)	20 (23.5)
Couple of times a month	11 (33.3)	15 (17.6)	14 (16.5)
Couple of times a week	7 (21.2)	5 (5.9)	8 (9.4)
Daily	2 (6.1)	6 (7.1)	4 (4.7)

**Table 2:** Climbing Details.

	Leisure & novice (N = 33)	Moderately experienced (N = 85)	Experienced (N = 85)	Total (N = 203)
Primary location				
Outdoors	1 (3.0%)	16 (18.8%)	34 (40.0%)	51 (25.1%)
Indoors	26 (78.8%)		61 (71.8%)	35 (41.2%) 122(60.1%)
No Difference	6 (18.2%)	8 (9.4%)	16 (18.8%)	30 (14.8%)
Most frequent type				
Top-Roping	7	16	13	36
Lead Climbing	3	27	59	89
Bouldering	28	64	44	136
Regularity (per week)				
Less than once	24 (72.7%)	37 (43.5%)	15 (17.6%)	76 (37.4%)
Once	5 (15.2%)	18 (21.2%)	26 (30.6%)	49 (24.1%)
Twice	2 (6.1%)	17 (20.0%)	23 (27.1%)	42 (20.7%)
> Twice	2 (6.1%)	13 (15.3%)	19 (24.7%)	34 (16.8%)

A study published in 2014^27^ explicitly informs the categorization used in this study, distinguishing four levels of climbing experience. Participants with less than one month of experience are considered leisure climbers (n = 8), and participants with more than one month but less than one year of experience are considered novice climbers (n = 25). Participants with one to four years of experience are moderately experienced climbers (n = 85), and participants with more than five years of experience are considered experienced climbers (n = 85).

### Procedure

Data were collected between December 22, 2022**,** and February 7, 2023. Participants were recruited through social media platforms, such as Facebook groups, and flyers distributed in climbing gyms around Regensburg, Bavaria, and at the University of Regensburg. The flyer, “Living on the Edge? Answer some questions for science,” featured a QR code and an image of a person climbing in the background. Notably, mindfulness was not mentioned in the flyer. The study, including all methods and hypotheses, was pre-registered on the Open Science Framework (https://osf.io/hr26y/?view_only=deeadd6373b2438ba196bb446a35b15e) before data collection. The survey was created using SoSci Survey**,** version 3.7.02^44^. The study adhered to the ethical standards outlined in the 1964 Declaration of Helsinki and followed the relevant guidelines and regulations. Informed consent was obtained from participants before answering the questionnaire. Ethical approval for this study was not required (see additional information).

After agreeing to the informed consent and terms of data protection, participants answered 118 items in total, which took about 10 min. After answering questions on socio-demographic information and details about their climbing experience, participants responded in the following order: questionnaires measuring non-attachment, body awareness, attention regulation, mindfulness, emotion regulation, details about their meditation experience, and finally, their level of understanding of the English language.

### Measures

To assess participants’ socio-demographic background, questions were included regarding age, gender, native language, and mental health disorders. Participants between 18 and 60 years old, with a sufficient understanding of the English language, were included. We set the age limit to around 60 years to minimize the presence of many older participants in the group of experienced climbers and to avoid a further age gap between the three climbing groups. Nevertheless, 60 years has been chosen arbitrarily. At the end of the survey, participants were asked to rate their knowledge of English on a scale from 1 (very good: I understood almost everything) to 5 (not good: I do not understand English). Four questions were included about participants’ climbing backgrounds. Because there is some evidence that physical activity in natural environments affects levels of mindfulness, the primary location of climbing (outdoors / indoors / no difference) was assessed to control for effects^45^. Additionally, it was asked how long their experience in climbing was in years, how frequently they were climbing or bouldering in the past half year, and in which type of climbing they participated most frequently (top-roping/ lead climbing/ bouldering).

To describe the sample in more detail, previous experiences of meditation (“I have never meditated, I just tried it once, I meditate a couple of times a year, I meditate a couple of times a week, I meditate daily”) (see Table 1) or mindful sport (Yoga, Tai Chi, QiGong) was assessed.

The Five Facet Mindfulness Questionnaire (FFMQ) was used^46^ to assess mindfulness in climbers. The questionnaire consists of 39 items, answered on a 5-point Likert scale, ranging from 1 (never or very rarely true) to 5 (very often or always true). Five facets are calculated: *observing*, *non-reactivity to inner experience*, *acting with awareness*, *non-judging of inner experience*, and *describing*. *Observing* describes the ability to notice and attend to internal or external experiences, for example, sensations, emotions, cognitions, sounds, or smells. (Example item: “*When I am walking, I deliberately notice the sensations of my body moving”). Describing* is the ability to define internal experiences in words (Example item: “*I’m good at finding words to describe my feelings”). Acting with awareness* refers to the ability to attend to present-moment activities instead of acting automatically with the understanding of something different (autopilot) (Example item reversed: “*I’m easily distracted”)*. The *non-judging of inner experience* subscale assesses the ability to take a non-evaluative perspective on one’s thoughts and feelings (Example item reversed: “*I tell myself I shouldn’t be feeling the way I’m feeling”)*. The subscale *non-reactivity to inner experience* captures the ability to let one’s thoughts and feelings arise and pass without being overly distracted by them (Example item: “*In difficult situations, I can pause without immediately reacting”)*. Higher scores indicate the heightened presence of the constructs. Since the calculation of the total score was validated, the total score was also calculated accordingly. In this present study, Cronbach’s alpha of the subscales ranged from ɑ = 0.73 to ɑ = 0.93, and McDonald’s Omega ranged between ω = 0.72 (*observing*) and ω = 0.93 (*non-judging*). Item 11 was removed from the subscale *observing* due to insufficient selectivity, indicated by an inter-item correlation below 0.30^48^. Eight items of the total score showed insufficient selectivity, indicated by inter-item correlations below 0.30. Therefore, the total score of the FFMQ could not be interpreted^47^.

Because this study lacks a comparison group of non-climbers, Table 4 presents an overview of the descriptive data for the different subscales of mindfulness measured with the FFMQ compared to two other German samples. Those samples were chosen because the participants came from the same region where the flyers of this study were distributed. In the first comparison study^48^, participants ranged in age from 18 to 65 (mean age: 27.82), with 71% identifying as female. The second comparison sample^49^ consisted of Applied Movement Science students with a mean age of 22 years and 65% female representation. The two samples were younger and included a higher proportion of women. However, the total scores on the FFMQ were comparable to those in the present sample.

Attentional abilities were assessed using the Attentional Control Scale (ACS)^50^. The ACS comprises two subscales: *Attention Focusing and Attention Shifting*. *Focusing* describes the capacity to intentionally sustain attention on a desired channel and resist unintentional shifting to irrelevant or distracting objects (Example item: “My concentration is good even there is music in the room around me”). *Shifting* measures the capacity to purposely change the focus of attention to a desired object (Example item: “It is easy for me to alternate between two different tasks”). Both subscales consisted of seven and eight items rated on a 4-point scale ranging from 1 (almost never) to 4 (always). Higher scores represent a better ability to regulate attention. In this study, the internal consistency of the total score reached ɑ = 0.83, and for the subscales, Cronbach’s alpha ranged from ɑ = 0.81 (*focusing*) to ɑ = 0.75 (*shifting*). McDonald’s Omega indicated similar reliability: *shifting*: ω = 0.74, *focusing*: ω = 0.80, and for the total score: ω = 0.82.

Emotion regulation abilities were assessed using the Brief Version of the Difficulties in Emotion Regulation Scale (DERS-16)^51^. This scale was selected because emotion regulation, unlike constructs such as agency and sensation seeking, is strongly related to mindfulness^7^, and previous research has demonstrated a high correlation between the DERS and the FFMQ^51^. The questionnaire comprises 16 items rated on a 5-point Likert scale ranging from 1 (almost never) to 5 (almost always). The questionnaire assesses five subscales: *Lack of emotional clarity* (*clarity*) (Example item: “I am confused about how I feel”), *difficulties engaging in goal-directed behavior* (*goal*) (Example item: “When I’m upset, I have difficulty getting work done”), *impulse control difficulties* (*impulse*) (Example item: “When I’m upset, I feel out of control”), *limited access to effective emotion regulation strategies* (*strategies*) (Example item: “When I’m upset, I start to feel very bad about myself”), and *non-acceptance of emotional responses* (*non-acceptance*), (Example item: “When I’m upset, I become irritated with myself for feeling that way”). Higher scores indicate more problems in emotion regulation. In this study, the internal consistency of the total score was excellent with ɑ = 0.93. Cronbach’s alpha for the subscales ranged from ɑ = 0.75 to ɑ = 0.86. McDonald’s Omega for the total score was ω = 0.93. For the subscales it ranged from ω = 0.82 to ω = 0.85. For the subscale *clarity*, McDonald’s Omega could not be calculated because there were only two items.

Body awareness and moment-to-moment experiences were assessed using the Embodied Mindfulness Questionnaire (EMQ)^52^. The EMQ consists of 24 items with 5 subscales, each rated on a 5-point Likert scale ranging from 1 (almost never, [0–10%]) to 5 (almost always [91–100%]). *Detachment from automatic thinking* is the ability to disengage from unintentional thinking or automatic thinking patterns, considered a foundational skill for other embodied mindfulness abilities (Example item reversed: “I get absorbed by my thoughts”). *Attention and awareness of feelings and bodily sensations* measure maintaining attention and awareness of emotional and physical sensations throughout the body (Example item: “I notice my physical sensations”). *Disconnection with the body* measures the capacity to sustain bodily awareness over time, resulting in a continuous sense of connection (Example item: “I feel detached from my body”). *Awareness of the mind–body connection* refers to the understanding of the reciprocal relationship between mental and physical states (Example item: “I notice how my negative thoughts impact my mood”). *Acceptance of feelings and bodily sensations* measures the ability not to distract oneself or avoid one’s emotions or bodily sensations (Example item reversed: “I try to escape negative feelings”). In this study, the internal consistency for the subscales was good to excellent, with Cronbach’s alpha ranging from ɑ = 0.76 to ɑ = 0.94, McDonald’s Omega values ranged from ω = 0.76 to ω = 0.94 in this sample.

*Non-attachment* was assessed using the Short Form of the Non-Attachment Scale (NAS-SF)^53^. The scale measures a construct rooted in the Buddhist understanding of insight into the impermanent nature of mental representations, typically cultivated through meditation. *Non-attachment* is a subjective quality characterized by a relative absence of fixation on thoughts, images, or sensory experiences and a lack of internal compulsion to obtain, retain, avoid, or modify circumstances or experiences. The questionnaire consists of eight items *(Example item: I can let go of regrets and feelings of dissatisfaction about the past”)* scored on a 6-point Likert scale ranging from 1 (disagree strongly) to 6 (agree strongly). Higher scores reflect a greater presence of *non-attachment*. In this present study, item 6 was removed due to poor selectivity. The internal consistency for the adapted scale was acceptable, with Cronbach’s alpha ɑ = 0.76, and McDonald’s Omega of ω = 0.76 in this sample.

### Data analysis

Data were analyzed using SPSS Version 29.0^54^. Mean scores were calculated to obtain the total and subscale scores for each of the five questionnaires. Climbing experience groups were created based on predefined cut-off scores. Due to unequal group sizes, leisure and novice climbers were combined into a single group (n = 33) for subsequent analyses. Descriptive statistics were computed for demographic variables and all dependent measures. In fifteen cases, participants failed to complete one or two of the five questionnaires fully. These instances were treated as missing values, a deviation from the original pre-registered plan.

To test the first hypothesis, a MANOVA was conducted with three climbing groups (leisure and novice climbers, moderately experienced climbers, and experienced climbers) and five facets of the FFMQ (*acting with awareness, non-judging, describing, and non-reactivity*). A separate ANOVA was also performed for the *observing* subscale, as it did not correlate significantly with the other subscales of the FFMQ. For the second hypothesis, a MANOVA was conducted with the three climbing groups and two subscales of the ACS as dependent variables. Furthermore, a separate ANOVA was calculated for the total ACS score. A MANOVA with three climbing groups and the five correlated subscales of the DERS-16 scale was performed for the third hypothesis. An additional ANOVA was conducted for the total DERS-16 score. The assumption of homogeneity of covariance (Box’s M test) and homoscedasticity of error variance (Levene’s test) was met, as both tests were insignificant. However, the assumption of normal distribution was violated for some dependent variables, as indicated by a significant Shapiro–Wilk test. Following the recommendations by Finch^55^ and Blanca et al.^56^, MANOVA and ANOVA are relatively robust against violating this assumption.

In a sensitive analysis, the participants were categorized based on their meditation experience into two groups: those with less experience, including individuals who meditated only a few times per year, and those with more extensive knowledge, comprising participants who reported meditating monthly, weekly, and daily. The leisure and novice athletes’ group includes more athletes with more meditation experience than the other two groups, which did not differ from each other, χ2 (2, 203) = 10.88, *p* = 0.004, see Table 1. This factor, meditation experience, was included in an added analysis for each hypothesis.

For another exploratory, not preregistered analysis, multivariate regression analyses were conducted. Gender was included as an independent variable, while meditation practice assessed on a 6-point scale ranging from 1 (never) to 6 (daily), age, climbing experience measured on a 10-point scale ranging from 1 *(*less than a month) *to 10 (*more than ten years*),* and the interaction between meditation experience and climbing experience were added as covariates. The interaction term was included to account for differences in meditation experience across the three climbing groups. For each of the six single regressions that are included in the multivariate regression, there was no autocorrelation (Durbin-Watson statistics around 2) and multicollinearity, which was measured with the variation inflation factor (VIF was < 5.1) and tolerance, the reciprocal of VIF, was < 1.0.

## Results

The means and standard deviations of all the dependent variables are presented in Tables 3 and 4,

**Table 3:** Description of the dependent variables.

	Leisure & novice (N = 33)		Moderately experienced (N = 85)		Experienced (N = 85)		Total (N = 203)	
	*M*	*SD*	*M*	*SD*	*M*	*SD*	*M*	*SD*
ACS total	2.63	.43	2.57	.47	2.66	.46	2.62	.46
Focusing	2.74	.47	2.65	.55	2.67	.53	2.67	.57
Shifting	2.52	.53	2.51	.53	2.64	.52	2.56	.52
DERS total	2.03	.71	2.36	.69	2.02	.72	2.16	.73
Clarity *	1.89	.73	2.17	.77	1.83	.67	1.98	.73
Goals	2.76	.88	2.89	.86	2.65	.95	2.77	.90
Impulse	1.80	.86	1.87	.93	1.79	.84	1.83	.88
Strategies *	1.92	.82	2.35	.88	1.90	.80	2.09	.86
Nonacc *	1.84	.81	2.46	1.00	2.02	.98	2.17	1.00
EMQ								
Detach	2.65	.56	2.57	.54	2.73	.68	2.69	.61
Awafeel	3.80	.62	3.80	.63	3.87	.61	3.83	.62
Disconn	1.89	.84	1.76	.71	1.65	.71	1.74	.73
Awamindbody	3.78	.77	3.69	.73	3.57	.87	3.66	.80
Accfeel	2.90	.79	2.91	.68	2.92	.70	2.91	.70
NAS total *	4.39	.71	4.15	.72	4.42	.71	4.30	.72

Detach = Detachment from Automatic Thinking, Awafeel = Attention and Awareness of Feelings and Bodily Sensations, Disconn = Connection with the body, Awamindbody = Awareness of the Mind–Body Connection, Accfeel = Acceptance of Feelings and Bodily Sensations. The * indicates a significant difference (*p* < .05) in the variable between the three climbing groups*.*

**Table 4:** Description of the mindfulness values compared to two other samples, non-climbers, in the same country.

	Leisure & Novice (N = 33)		Moderately Experienced (N = 85)		Experienced (N = 85)		Total		(Schroter et al., 2022)^55^		(Siebertz et al., 2022)^56^	
	*M*	*SD*	*M*	*SD*	*M*	*SD*	*M*	*SD*	*M*	*SD*	*M*	*SD*
FFMQ total	3.40	.47	3.33	.47	3.47	.45	3.35	.48	3.46	.44	3.40	.45
Observe	3.67	.61	3.58	.56	3.53	.59	3.50	.62	3.51	.60	3.57	.58
Nonreac	3.06	.66	3.05	.62	3.15	.55	2.99	.66	3.13	.52	3.90	.56
ActAwa	3.34	.69	3.16	.63	3.34	.76	3.30	.67	3.34	.73	3.28	.68
Nonjudge *	3.53	.75	3.32	.86	3.80	.91	3.44	.83	3.81	.92	3.57	.88
Describe	3.34	.87	3.52	.82	3.50	.78	3.47	.74	3.47	.79	3.47	.82

FFMQ = Five-Facet Mindfulness Questionnaire, Nonreact = Non-Reacting, ActAwa = Acting with Awareness, Nonjudge = Non-Judging; The * indicates a significant difference (*p* < .05) in the variable between the three climbing groups.

### Dispositional mindfulness

Regarding the investigation of mindfulness (Hypothesis 1), the MANOVA using Pillai’s-trace indicated a statistically significant overall difference between the climbing groups (*F*(8, 388) = 2.147, *p* = 0.031, η^2^ = 0.042). However, the climbing groups only differ in the subscale *non-judging* (*F*(2, 196) = 6.434, *p* = 0.002, η^2^ = 0.062). Tukey HSD post-hoc comparisons indicated that moderately experienced climbers scored significantly lower on *non-judging* than experienced climbers (*p* = 0.001, *M*_*Diff*_ = -0.48, 95% CI [− 0.79, − 0.16]). There were no statistically significant group differences between the experience levels for the subscales *non-reacting* (*F*(2, 196) = 0.64, *p* = 0.529, η^2^ = 0.006), *acting with awareness* (*F*(2, 196) = 1.873, *p* = 0.156, η^2^ = 0.019) and *describing* (*F*(2, 196) = 0.591,* p* = 0.555, η^2^ = 0.006). Additionally, no significant differences were observed for the subscale *observing* (*F*(2, 196) = 0.73, *p* = 0.481).

Exploratory analyses additionally examined the meditation experience (categorized as less vs. more experience) as a factor. Although higher scores were found for the subscales *observing* and *non-reactivity* in participants with more meditation experience, these differences were independent of climbing group membership, as shown by the lack of a significant interaction effect between meditation experience and climbing group on mindfulness aspects. All means and standard deviations are presented in Table 4.

### Attention regulation

Concerning attention regulation (Hypothesis 2), the ANOVA for the total score of the ACS, indicated no significant differences between the climbing groups (*F*(2, 200) = 0.80, *p* = 0.447, η^2^ = 0.008). A MANOVA (Pillai’s-trace) was conducted with three climbing groups and two measurements corresponding to the ACS subscales. Again, no statistically significant differences between the climbing groups were observed for the combined dependent variables (*F*(4, 400) = 1.38, *p* = 0.242, η^2^ = 0.01). Exploratorily, the meditation experience (less experience, more experience) was investigated as an additional factor. However, no significant main effect of meditation experience, nor a significant interaction between meditation experience and climbing group, was found regarding attention control.

### Emotion regulation

For the third hypothesis concerning emotion regulation, an ANOVA using the total score of the DERS-16 revealed a significant difference between the three climbing groups (*F*(2, 197) = 5.12, *p* = 0.007, η^2^ = 0.049). Tukey HSD post-hoc analysis indicated that moderately experienced climbers had significantly higher total scores than experienced climbers (*p* = 0.009, *M*_*Diff*_ = 0.33, 95% CI [0.07, 0.59]). The other pairwise comparisons were not significant. Exploratory, meditation experience (less vs. more experience) was examined as an additional factor. No significant main effects of the meditation experience or the interaction between the meditation experience and the climbing group were found for emotion regulation.

The MANOVA (Pillai’s-trace) revealed significant differences between the climbing groups across the *emotion regulation* subscales (*F*(10, 388) = 2.74, *p* = 0.003, η^2^ = 0.07). Tests of between-subject effects showed no statistically significant differences between the experience levels for the subscales *goals* (*F*(2, 197) = 1.491,* p* = 0.228, η^2^ = 0.015) and *impulse* (*F*(2, 197) = 0.233,* p* = 0.792, η^2^ = 0.002). However, significant differences between the climbing groups were found for the subscales *clarity* (*F*(2, 197) = 5.12, *p* = 0.007, η^2^ = 0.05), *strategies* (*F*(2, 197) = 6.93, *p* = 0.001, η^2^ = 0.07) and *non-acceptance* (*F*(2, 197) = 6.81, *p* = 0.001, η^2^ = 0.07).

Tukey HSD post-hoc analysis revealed the following significant group differences: For *clarity*, moderately experienced climbers had significantly higher scores than experienced climbers (*p* = 0.006, *M*_*Diff*_ = 0.35, 95% CI [0.08, 0.61]). For *strategies*, it was shown that moderately experienced climbers had significantly higher scores than leisure and novice climbers (*p* = 0.035, *M*_*Diff*_ = 0.43, 95% CI [0.02, 0.83]) and experienced climbers (*p* = 0.002, *M*_*Diff*_ = 0.45, 95% CI [0.14, 0.76]), for *non-acceptance* the results demonstrated that leisure and novice climbers had significantly lower scores than moderately experienced climbers (*p* = 0.006, *M*_*Diff*_ = − 0.63, 95% CI [− 1.10, − 0.15]). Furthermore, moderately experienced climbers had significantly higher scores than experienced climbers (*p* = 0.009, *M*_*Diff*_ = 0.45, 95%-CI [0.09, 0.81]). All other comparisons were not statistically significant.

We explored the experience of meditation as another factor (less vs. more experience). There was no significant effect for one of the subscales and no interaction effect between the mediation experience and the climbing group.

### Body awareness and non-attachment

The multivariate regression analysis revealed significant effects for meditation practice (*F*(6, 188) = 4.835, *p* < 0.001, η^2^ = 0.134), the interaction between meditation practice and climbing experience (*F*(6, 188) = 2.434, *p* = 0.027, η^2^ = 0.072 and gender (*F*(6, 188) = 6.273, *p* < 0.001, η^2^ = 0.167).

Meditation practice predicted *attention and awareness of feelings and bodily sensations* (ß = 2.136, *p* = 0.016), *awareness of the mind body connection* (ß = 3.826, *p* = 0.008), the *acceptance of the feelings and bodily sensations* (ß = 2.634, *p* = 0.019), and *non-attachment scale* (ß = 2.927, *p* = 0.014). The interaction between meditation practice and climbing experience was a significant predictor for the detachment from automatic thinking (ß = 2.206, *p* = 0.014), *awareness of the mind–body connection* (ß = 2.372, *p* = 0.035), and *non-attachment scale* (ß = 2.850, *p* = 0.016). Gender was a significant predictor for *disconnection with the body* (ß = 4.007, *p* = 0.004) and *awareness of the mind–body connection* (ß = 16.653, *p* < 0.001), with higher values for women and with higher values for men for *acceptance of feelings and bodily sensations* (ß = 2.506, *p* = 0.022).

## Discussion

The study’s findings suggest that the relationship between various aspects of dispositional mindfulness and climbing experience is relatively weak: The first three hypotheses were only partly supported. Among the facets of dispositional mindfulness, only *non-judging* was significantly higher in experienced climbers than in moderately experienced climbers. The second hypothesis-that climbers with greater experience would demonstrate better attention control-was not supported. Regarding emotional regulation strategies, the results partly supported the third hypothesis. Specifically, moderately experienced climbers showed greater difficulties with emotional clarity and were less effective at accepting their emotions compared to novice and leisure climbers and experienced climbers. They also demonstrated less effective use of emotion regulation strategies than experienced climbers. A significant interaction between mindfulness and climbing experience was found concerning *detachment from automatic thinking*, *awareness of the mind–body connection*, and *non-attachment.*

Regarding dispositional mindfulness, the results are partly consistent with the findings of Gabriano et al.^57^, who also did not observe higher levels of mindfulness among more skilled boulders. However, in the present study, experienced climbers scored higher on the *non-judging* subscale. This may suggest that extensive climbing experience supports a more non-judgmental attitude toward one’s own thoughts and emotions. No significant differences were found between the experienced and novice and leisure groups. This could be explained by a higher prevalence of meditation within the latter group. Furthermore, a descriptive comparison with other data on dispositional mindfulness—collected using the same assessment tool in the same country- does not indicate that climbers generally exhibit higher values in dispositional mindfulness.

Regarding emotion regulation, the results are partly consistent with findings of Woodman et al.^36^, who showed that only climbers, compared to low-risk athletes, could transfer their experiences of agency and emotion regulation into their everyday lives. Based on the agentic emotion regulation perspective by Barlow et al.^32^, they concluded that regular climbing provides participants with an agentic emotional experience that benefits their daily functioning. What should be considered- and may align with the findings—is that individuals with emotional difficulties, as seen in studies on alexithymia, appear more drawn to high-risk sports^58^. It is likely that people facing greater challenges in emotion regulation, such as people with major depression, may use climbing as a strategy to better cope with emotions^59^.

In the present study, moderately experienced climbers tended to exhibit greater difficulties in emotion regulation. This supports the hypothesis that more experienced climbers have better emotion regulation skills than moderately experienced climbers. However, it contradicts the assumption that moderately experienced climbers would outperform novices in this regard. Willegers et al.^60^, in their study on agentic emotion regulation in climbers, distinguished—among others—between traditional rock climbers, reported to range from intermediate to expert (comparable to moderately experienced climbers in this study), and sport climbers (comparable to leisure and novice climbers here), who identified sport climbing as their preferred discipline. They investigated emotion regulation in both groups by asking participants to imagine being unable to engage in their sport for one day, one week, and six weeks. At the beginning of the study, there were no differences in emotion regulation between the two groups, which aligns with the findings presented here. However, after one and six weeks, traditional climbers showed increased difficulties in emotion regulation, whereas sport climbers did not. Although these results cannot be directly compared to those of the present study, they contribute valuable insight into the different possible scenarios for investigating emotion regulation in climbers.

Future investigations should further explore these aspects of emotion regulation. It would be interesting to examine whether the effects of climbing on emotion extend beyond those already established for regular exercise^61^. Including a control group of regular and non-exercisers would have provided more profound insight into the climbing-specific effects observed in this study. Moreover, to ensure comparability of results regarding emotion regulation in climbers, it is essential to apply the standardized grading scales, climber classifications, and ability grouping proposed by the International Rock-Climbing Research Association, IRCRA^62^

Unlike findings in other sports disciplines, where executive and attentional functions tend to be enhanced in more experienced athletes, this does not appear to be the case for climbers, as suggested by the present study^12,63^. However, it is crucial to note that previous studies primarily compared athletes to non-athletes. In light of the current findings, it must be acknowledged that attentional functions are multifaceted and that executive and cognitive functioning only partially capture attentional capabilities, complicating direct comparisons with earlier research.

Although previous studies have demonstrated that bodily awareness and control are crucial for continuous improvement in sports^64^, this study’s results show only a marginal relationship between climbing experience and one subscale of the body awareness measurement. This finding warrants further investigation; for instance, interoceptive ability could be assessed using non-questionnaire methods such as the heartbeat-tracking task, which measures the accuracy of perceiving and counting one’s heartbeats over a defined period.

One limitation of this study is the absence of a non-climbing control group. Including a control group of lightly active individuals and a group engaged in other forms of exercise would have provided a broader context for interpreting the results. It remains unclear whether the long-term participation in climbing observed in this study produces effects beyond those of other physical activities, and whether such effects can be attributed explicitly to unique aspects of climbing, such as its inherent risk.

We compared the results for dispositional mindfulness with two samples from the same regions^48,49^, one of which consisted of physically active students. The participants in these two comparison samples were slightly younger and had a higher proportion of women. Nevertheless, the scores were comparable across the groups. One limitation is that this study could not analyze the total mindfulness score in more detail due to its low internal consistency.

It should also be noted that more detailed information about the participants’ climbing backgrounds is necessary for a more accurate analysis. This study did not assess the specific type of climbing practiced, individual climbing ability, the consistency of climbing participation over time, or the recency of participants’ last climbing activity. Future research should incorporate ability groupings and adopt the classification system proposed by the IRCRA^62^. Another limitation was the relatively small sample size in the leisure and novice climbers’ group, which also reported a higher level of meditation experience, potentially influencing the results. Additionally, the study did not control for engagement in other high-risk sports, such as base jumping or mountaineering, nor did it account for personality traits like sensation seeking and alexithymia, which have been previously linked to climbing behavior. Also, the age limit was chosen arbitrarily, though it was intended that no geriatric climbers participated.

A third limitation is the low response rate to the questionnaire. One possible reason is that the sample and context may not have conveyed a clear personal benefit to the athletes, and no incentives were provided. However, as Wu et al.^65^ suggested, personal interaction or follow-up phone calls can help increase response rates and should be considered in future research. Additionally, the length of the questionnaire may have burdened participants. A further limitation is that all data were collected through self-report measures, susceptible to biases such as social desirability^66^ and acquiescence^67^.

Previous research has demonstrated the beneficial impact of exercise on emotional regulation, including its psychological and biological mechanisms^61^. In the context of the present study, it is important to explore whether the positive effects of climbing on emotion regulation go beyond those of other forms of exercise. Further investigation of the Agentic Emotion Regulation Theory by Barlow et al.^32^ could also provide valuable insight. Future research in high-risk sports domains should consider incorporating agency and sensation-seeking parameters. However, the current study did not address these aspects, as the primary focus was on the relationship between mindfulness and emotion regulation.

The findings of this study might also be interesting for therapeutic climbing, which has recently gained increased attention, particularly in treating patients with depression^68^. This study highlights the inherent potential of climbing to support emotion regulation and encourage a non-judgmental attitude toward one’s thoughts and experiences, even without therapeutic guidance. While a meta-analysis found statistically significant benefits of climbing only in the physical domain, the present study suggests that a greater degree of climbing experience may be required to impact emotional well-being^68^. This aligns with findings from Gürer et al.^69^, who reported that professional adolescent rock-climbers exhibited lower anxiety levels compared to non-climbers. These results indicate that a higher frequency or intensity of climbing activity may be necessary to achieve mental health benefits. Further interventional studies are needed to explore potential causal relationships between climbing activity, emotion regulation, and the psychological factors examined in this study. Future research should also consider additional variables, such as sensation-seeking. For example, Rumbold et al.^37^ found that healthy climbers scored higher on specific sensation-seeking subscales than those with emotional instability. Moreover, multicenter and longitudinal experimental studies are essential to advancing our understanding of mindfulness in the context of sports.

To conclude, this study shows that the experience of climbing is only related to the non-objective facet of dispositional mindfulness. The observed advantages in emotion regulation among experienced climbers suggest that sustained engagement in climbing is associated with an agentic emotional experience. Regarding the variables of body awareness, non-attachment, and detachment from automatic thinking, a significant interaction was found between mindfulness and climbing experience.

## Data Availability

The data can be retrieved from osf: https://osf.io/hr26y/?view_only=deeadd6373b2438ba196bb446a35b15e
